# Population Analysis and Evolution of *Saccharomyces cerevisiae* Mitogenomes

**DOI:** 10.3390/microorganisms8071001

**Published:** 2020-07-04

**Authors:** Daniel Vieira, Soraia Esteves, Carolina Santiago, Eduardo Conde-Sousa, Ticiana Fernandes, Célia Pais, Pedro Soares, Ricardo Franco-Duarte

**Affiliations:** 1 Centre of Molecular and Environmental Biology (CBMA), Department of Biology, University of Minho, 4710-057 Braga, Portugal; jdanav@gmail.com (D.V.); soraia-aires@hotmail.com (S.E.); carolina.santiago.t@hotmail.com (C.S.); econdesousa@gmail.com (E.C.-S.); tixand.tf@gmail.com (T.F.); cpais@bio.uminho.pt (C.P.); pedrosoares@bio.uminho.pt (P.S.); 2 Institute of Science and Innovation for Bio-Sustainability (IB-S), University of Minho, 4710-057 Braga, Portugal; 3 CMUP—Centro de Matemática da Universidade do Porto, 4169-007 Porto, Portugal

**Keywords:** mitochondrial genome, mitogenome, recombination, *Saccharomyces cerevisiae*, yeasts, genomic characterization

## Abstract

The study of mitogenomes allows the unraveling of some paths of yeast evolution that are often not exposed when analyzing the nuclear genome. Although both nuclear and mitochondrial genomes are known to determine phenotypic diversity and fitness, no concordance has yet established between the two, mainly regarding strains’ technological uses and/or geographical distribution. In the current work, we proposed a new method to align and analyze yeast mitogenomes, overcoming current difficulties that make it impossible to obtain comparable mitogenomes for a large number of isolates. To this end, 12,016 mitogenomes were considered, and we developed a novel approach consisting of the design of a reference sequence intended to be comparable between all mitogenomes. Subsequently, the population structure of 6646 *Saccharomyces cerevisiae* mitogenomes was assessed. Results revealed the existence of particular clusters associated with the technological use of the strains, in particular regarding clinical isolates, laboratory strains, and yeasts used for wine-associated activities. As far as we know, this is the first time that a positive concordance between nuclear and mitogenomes has been reported for *S. cerevisiae*, in terms of strains’ technological applications. The results obtained highlighted the importance of including the mtDNA genome in evolutionary analysis, in order to clarify the origin and history of yeast species.

## 1. Introduction

*Saccharomyces cerevisiae* is a microorganism with great importance for humanity, having been used for centuries in day-to-day activities [1]. This yeast has a relevant role in the study of cellular and biochemical pathways, having the capacity to obtain energy solely by fermentation, making it a facultative anaerobe [2,3,4].

In 1996, the 12 Mb *S. cerevisiae* genome was the first eukaryotic nuclear genome to be sequenced [1,5], and it is arguably the most thoroughly annotated todays [6]. *S. cerevisiae* is also one of the most studied microbes, often used as a model organism owing to its short generation time, the ability to control its sexual cycle, and the availability of tools and knowledge for genomic manipulation [7,8], prompting its use in biotechnology applications. The mitochondrion, also known as the “powerhouse” of the cell, is a fundamental organelle enclosed by two lipid membranes [9,10]. Mitochondria are essential for cellular respiration and contain their own genome, the mitochondrial DNA (mtDNA) or the mitogenome, independent of the nuclear genome. The mitochondria is the result of an ancestral symbiont α-proteobacterium that conjugated with an early eukaryotic cell, followed by the transfer of genes from the mtDNA genome to the nucleus, leading to a significant reduction of the size of the mitochondrial genome [11,12]. Besides being metabolic mediators, mitochondria also take part in several cellular functions such as proliferation [13], ATP production, respiration, metabolite biosynthesis, and ion homeostasis [14]; are key players in programmed cell death [15]; and are involved in multiple signaling pathways [16,17,18]. Mitochondrial genomes are typically circular and double-stranded, and their DNA is more prone to undergoing mutations than the nuclear DNA [17]. Numerous copies of this genome can be found in every cell (between 50 and 200) [16], depending on various factors such as the tissue [19] or culture conditions [20,21].

Depending on the external conditions, yeast cells can be in haploid or diploid phase. Under stressful conditions, diploid cells carry out sporulation followed by meiosis, and four haploid spores are generated [22], resulting in the haploselfing [23]. Budding yeasts inherit mtDNA biparentally [24], as opposed to the majority of higher eukaryotes, for which mtDNA inheritance is uniparental [25]. After DNA recombination in zygotes, homoplasmy between diploid offspring is obtained, with only one mtDNA haplotype being kept [24]. In this way, all copies of the mtDNA are expected to be similar in a cell population after nearly 20 generations, since the tendency of the mitochondrial system is to maintain the cell population with homogeneous copies of mtDNA [26]. Therefore, the prediction of allele distribution is very difficult, due to the loss of heteroplasmy and also due to the lateral transference of mitochondrial mobile elements in populations [27]. Working from the consensus that genetic variation in mtDNA provides adaptive potential, it is necessary to understand the effects of this loss of heteroplasmy, and the factors that influence functional mtDNA variation.

Large rearrangements, accumulation of intergenic sequences, and point mutations have culminated in a high diversity in the structure and organization of *S. cerevisiae* mitochondrial genomes. Additionally, *S. cerevisiae* mitochondria move through cytoskeletal paths, fusing and dividing frequently [28,29,30], increasing the dynamics that generate diversity. When dividing between mother cell and bud, mitochondria rely on complex network actions in order to distribute the organelles evenly across the cells [31,32,33]. Replication and partitioning of the mitochondrion is not related to the cell cycle, depending instead on the replication and expression of its own genes and nuclear-encoded proteins [10,34]. For mitochondrial biogenesis, both nuclear and mitochondrially encoded components are required [10]. Since the mitogenome encodes a small subset of proteins, it requires the ones encoded in the nucleus in order to express its genome [35]. One important aspect to consider is that this interaction between the two genomes (nuclear and mitochondrial) allows for the determination of phenotypic diversity and fitness [10,16,34]. In this way, it was expected that the same type of population structure would be visible, in terms of strains’ technological uses and/or geographical distribution, as that detected for nuclear genomes alone [1,36]; however, this was not, surprisingly, the common scenario found until now.

Mitochondrial DNA recombination occurs following mating, during the transition from the heteroplasmic to homoplasmic phase, as mentioned above. This homologous recombination is expected to result in gene reorganization and enhanced selection on adaptive loci. Among cases of biparental mtDNA inheritance, mitochondrial recombination is common, as observed in fungi. High levels of reticulation and diversity among *Saccharomyces* mtDNAs reveal a path involving recombination and also horizontal gene transfer within and between species [24]. Mitochondrial recombination has been correlated with hybrid fitness, removal of deleterious mutations, and coevolutionary processes; it has been described in fungi [24], plants [37], protists [38], and invertebrates [39]. Several studies have reported differential mitochondrial alleles, or even entire mitotype selection depending on environmental changes. This fact suggests that mitochondrial recombination has an important impact on mito–mito epistasis with consequences for functional variation. Additionally, cellular homeostasis has been correlated with mito–nuclear interactions [24] indicating the important role of mtDNA and its adaptive potential. Although mitochondrial recombination has played a role in the evolution of mtDNA in yeasts [24,40], and high rates of recombination have been reported in laboratory conditions [41], a deep study focus on its association with the nuclear genome and with phenotypic diversity is lacking. Most studies have focused only on laboratory isolates, with phenotypic diversity shaped by laboratory manipulations, which was believed to lead to the “petite” type associated with the slow-growth phenotype observed in cells without mitochondrial genomes [42,43].

Recombination can take place over short repeats [44], or homologous pairing [45]. Although it was previously supposed that recombination produces molecules with different gene orders and/or intergenic deletions [46,47], it was recently suggested using whole-genome sequencing approaches that gene order is species-specific [48]. Hotspots are positions, typically within intergenic regions, in which recombination is most likely to occur, probably owing to repetitive and palindromic elements. Based on the information available to date, it is presumed that mitochondrial recombination usually takes place in non-protein-coding regions [49,50]. Intron homing is a mechanism through which mobile introns allow for easier recombination of their corresponding intron-containing genes. However, in the mitochondrial genome, the absence of introns does not necessarily imply either low nucleotide diversity or lack of recombination hotspots. Stand-alone endonucleases (SAEs) have been proposed by Wu et al. [51] to be the elements responsible for the recombination and high diversity of adjacent intron-lacking genes.

In 2014, Fritsch et al. [41] proposed a revolutionizing map of mtDNA recombination in *S. cerevisiae*, using a cross between two strains to detect recombination events, in contrast to available research using only reporter genes or artificial systems. Authors reported averages of 2.3 to 3 recombination events per kilobase, with the remarkable conclusion that recombination was very uneven along the genome map. However, only two *S. cerevisiae* strains were included in this analysis, and so no clear conclusions about strain diversity could be drawn. The objective of the present work was to assess population structure within *S. cerevisiae* isolates via mitochondrial DNA analyses, expanding the previously established recombination map of Fritsch et al., considering a large expansion in the number of strains, and relating the observed diversity with strains’ technological uses. Since the mechanism of mtDNA inheritance is peculiar in yeasts, being biparentally inherited in contrast to higher eukaryotes, in which mtDNA inheritance is uniparental, it is of great importance to clarify the effects of this process on the population structure of yeasts, and on the phenotypic diversity observed.

## 2. Materials and Methods

### 2.1. Dataset Collection

Mitochondrial DNA (mtDNA) genomes from *S. cerevisiae* were obtained from NCBI from two sources (data collected in October 2017): directly, as annotated nucleotide data, and from the Sequence Reads Archive (SRA) database, containing next-generation sequencing raw data files from genomic data (DNA) of *S. cerevisiae*.

We used the sequences deposited as nucleotide data in NCBI to define a common region of analysis that could be applied to the complete dataset. Complete mtDNA sequences were virtually impossible to align using common multiple-alignment software tools, for two reasons: one related to the extremely high content of adenine and thymine outside coding regions (almost 100%) which is not informative for the multiple alignment or for any evolutionary analysis; the second issue was related to the presence of different pseudogenes/genes in different mtDNA genomes.

In order to obtain a comparable dataset that could be used in evolutionary and population genetics’ analysis, we focused on protein-coding genes and, within these, we restricted our analyses to those present across all samples of our mtDNA dataset. We extracted a subset of protein-coding region segments from the *S. cerevisiae* reference sequence (*Saccharomyces cerevisiae* S288C; assembly R64) and performed a BLAST search for each of those protein-coding regions in the remaining lineages (in order to avoid missing data based on poor annotation of some mtDNA genomes). After compiling the data, we checked whether missed regions in specific samples were not detected due to missing data in specific sequences with lower quality.

The raw data from the SRA database were downloaded using the SRA toolkit (www.ncbi.nlm.nih.gov/sra/docs/toolkitsoft) and aligned against the reference genome using the Burrows–Wheeler Aligner (BWA) [52]. Next, bcftools (http://samtools.github.io/bcftools/) was used to convert file formats and extract the target regions defined above.

For each of the extracted sequences, whether nucleotide data or as raw genomic data, we inspected the database and associated literature when available for information on isolation source, geographical location, strain code, and collection date.

### 2.2. Alignment

MAFFT version 7 [53] was used to multiple-align the extracted regions from all the samples. Following the alignment, we performed quality control by removing all sequences that failed to display nucleotide data for more than 10% of the analyzed locations due to unread positions. The sequences remaining after this first step were submitted to IMPUTOR [54] in order to estimate/impute some of the remaining unread positions given the overall alignment and a maximum-likelihood phylogenetic reconstruction using MEGA7 [55], using a general time reversible (GTR) model with uniform rates. However, as discussed later, the high rate of recombination did not allow for the data to be analyzed phylogenetically. Nevertheless, this analysis indicated close similarity between sequences, as required for imputation and by the employed software.

### 2.3. General Statistics and Linkage Disequilibrium

The overall dataset (5475 bp) was used to estimate general statistics of the mtDNA data through DNAsp v.5.10 [56] and Arlequin [57], including nucleotide diversity, haplotype diversity, and number of haplotypes, as well as to perform general selection tests (Tajima’s D and Fu’s FS).

This dataset was also used to estimate recombination parameters and linkage disequilibrium (LD) measures. Data were filtered to include only SNPs with frequencies higher than 10%. LD measures (*D’* and *r*^2^) were calculated using Haploview [58]. Haplotype blocks were estimated using the standard parameters of the software. A second approach was the estimation of relative recombination parameters from SNP data using PHASE software [59] which ran for 100,000 iterations. The analysis was performed, establishing that the phase of the data was known (as homoplasic data was extracted from each organism individually), and considering the general model for recombination rate variation previously described [60] in the software.

### 2.4. Genetic Structure of S. cerevisiae mtDNA

Considering that the mtDNA in *S. cerevisiae* is highly recombinant, analysis should focus on distance and clustering algorithms instead of evolutionary phylogenetic analysis. We employed for this purpose three methodologies: principal component analysis (PCA), individual ancestry estimation, and a neighbor-joining (distance-based) phylogenetic analysis. Although some of these methods require independent SNPs (low LD), that level of independent variants was achieved for many variants in the *S. cerevisiae* mtDNA due to high recombination rates (discussed later). For these analyses, for simplicity, we focused only on samples for which isolation source and geography were known, and we aimed to check hypothetical clustering based on these parameters.

In order to cluster the different samples according to their genetic variation, we employed PCA, which estimates linear vectors that partially explain the observed variation. We used the standard PCA tool provided in EIGENSOFT v6.0.1 [61] to calculate the first 10 principal components (PCs) from which we calculated the fraction of variance. We used the three main vectors that explained the most diversity to build plots comparing each pair. Outlier samples in the analysis were removed for a second analysis so that we could focus on more detailed clustering patterns within the samples that were highly clustered on the first analysis. For another clustering method, we employed the neighbor-joining using MEGA7 [55]. While the dataset showed a great level of recombination, making it inappropriate for phylogenetic reconstruction, this method is distance-based, creating the possibility of establishing clusters (clades) of similar genotypes with low distance between them.

We estimated potential genetic components (K) in the data using sNMF [62], an algorithm for individual ancestry estimation. Individual ancestry estimation algorithms split the genetic data in each individual into predefined genetic components (Ks), which can be analyzed in a dataset by focusing on the sharing of the components between samples or on the presence of specific components in samples. We ran the analysis considering two to six probable components (K = 2 to K = 6).

Furthermore, we checked for population structure based on two predefined groups based on the geography of the samples and their source of isolation. We estimated general statistics for each group, and *Fst* between groups using DNAsp v.5.10 [56]. The obtained matrix of *Fst* values between groups was used to estimate a NJ tree using MEGA7 [55].

## 3. Results

### 3.1. Saccharomyces cerevisiae Mitochondrial Genome

**Figure 1:**
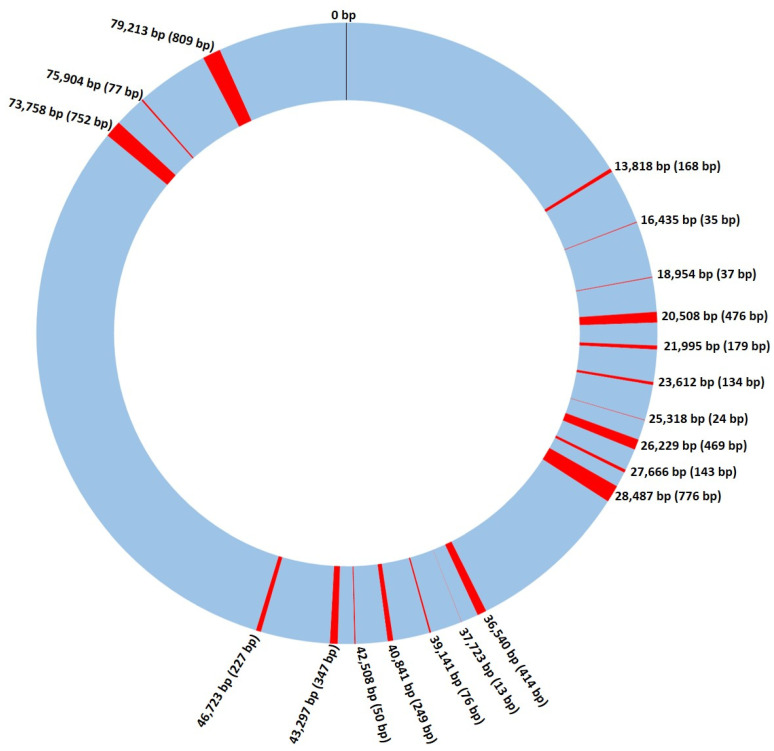
Schematic representation of the *Saccharomyces cerevisiae* mitochondrial genome. DNA regions used in the analysis shown throughout this work are marked in red, with the numbers representing its position on the genome (first base pair), and the numbers inside the brackets the size of each region. bp—DNA base pairs.

A total of 184 *S. cerevisiae* mitochondrial DNA sequences were retrieved from the NCBI nucleotide database (ncbi.nlm.nih.gov/nuccore; data collected in October 2017). These mitochondrial genomes were already fully annotated in terms of genes, proteins, and associated functions, and we used them to define a reference-comparable sequence between all mtDNA genomes to be applied in the subsequent analyses. Using this database, a common comparable portion of the mtDNA genome was compiled for all the samples in order to bypass the problems previously encountered when trying to align the retrieved sequences, mainly related to genome size and base and gene content. In this process, we defined the start and end positions of the mitochondrial genome to be used as the reference, together with the regions to be included. For this, we used the following strategy, as detailed in the Materials and Methods section: (i) only codifying regions were considered, since intergenic portions of the mitochondrial genome were revealed to be very variable in size and highly repetitive, contributing to an impossibility of alignment between the 184 genetic sequences; (ii) this non-codifying portion of the genome also had an extremely high amount of adenine and thymine (virtually 100%; data not shown) in terms of base composition, and because of this, it was excluded; (iii) a defined set of genes were included in the analyses since they were common to all specimens, but also to other species phylogenetically close to *S. cerevisiae*, highlighting their high conservation in yeast mtDNA. It is important to note that although the gene in general was conserved, the 3′ region of some genes often differed between strains. The analyzed portion of the reference sequence is illustrated in Figure 1 and it was used to study the larger group of samples in further analysis.

A total of 12,015 *S. cerevisiae* genomes were collected from SRA (Sequence Read Archive) section of NCBI (ncbi.nlm.nih.gov/sra; data collected in November 2017) as raw data (DNA) resulting from next-generation sequencing archives. The mitogenomes were extracted and aligned against the previously defined reference genome, mtDNA, from which the predetermined positions were extracted. Sequences with an excess (more than 10%) of unread nucleotide positions were excluded, the unread positions in the remaining mitogenome being determined with IMPUTOR [54].

A final dataset of 6646 genomes with a size of 5475 base pairs was obtained and submitted to DNAsp and Arlequin in order to obtain general statistics. Results are summarized in Table 1, and showed that even though the analysis was restricted to coding sequences and to a particular set of conserved genes, 72.28% of the final sequences was still composed of A (30.95%) and T (41.33%) nucleotides, respectively. This value was considerably smaller than the percentage of over 90% oberseved when considering the full mtDNA molecule, and that of virtually 100% outside the coding regions, but it clearly shows a biasing of the molecule towards these two bases.

One question that had to be addressed after using this smaller sequence was whether this segment provided enough diversity to render a good level of discrimination between sequences. Results showed that the diversity was high, with over 500 SNPs detected (Table 1). Considering the recombination effect, these 526 SNPs contributed to a very high haplotypic diversity (1265 different haplotypes) and a good discrimination between sequences (526 polymorphic sites and 38.63 pairwise differences, in average, between all sequences). There were also no signs of apparent selective pressure on the evolution of the *S. cerevisiae* mitochondrial genome (Tajima’s *D* and Fu’s *FS* respective *p*-values were not significant), which is a preferable feature in genetic systems when cataloguing diversity. However, this overall trend does not exclude that some particular haplotypes might have been related to specific features of the strains, as some researchers have already pointed out the selective potential of *S. cerevisiae* mtDNA [24].

### 3.2. Recombination and Linkage Disequilibrium in S. cerevisiae Mitochondrial DNA

**Figure 2:**
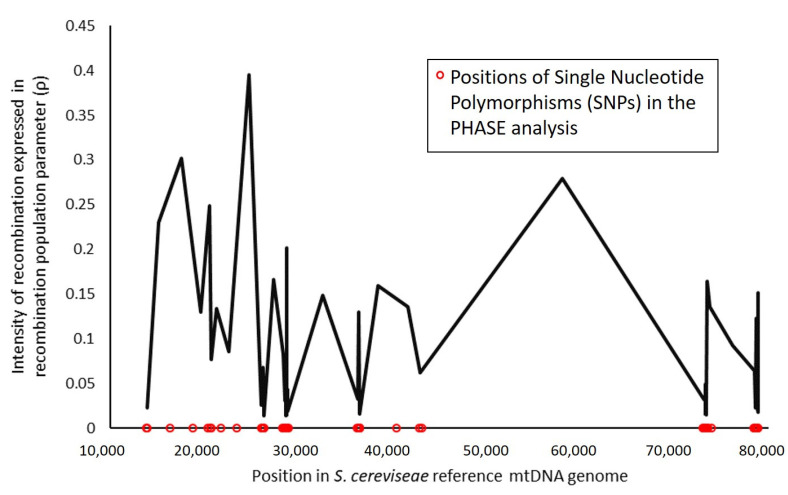
Intensity of recombination in the *Saccharomyces cerevisiae* mitochondrial genome in relation to an estimated background recombination rate, expressed in recombination population parameter.

In order to investigate recombination patterns across the *S. cerevisiae* mtDNA genome, two approaches were taken. Firstly, PHASE software was used to estimate recombination rates between polymorphisms. Results (Figure 2) showed a high intensity of recombination detected for the analyzed positions in the mtDNA molecule. Although the method is very sensitive for sequencing errors, it was evident that recombination in general is high across the entire mitochondrial genome and mostly prevalent in intergenic regions.

**Figure 3:**
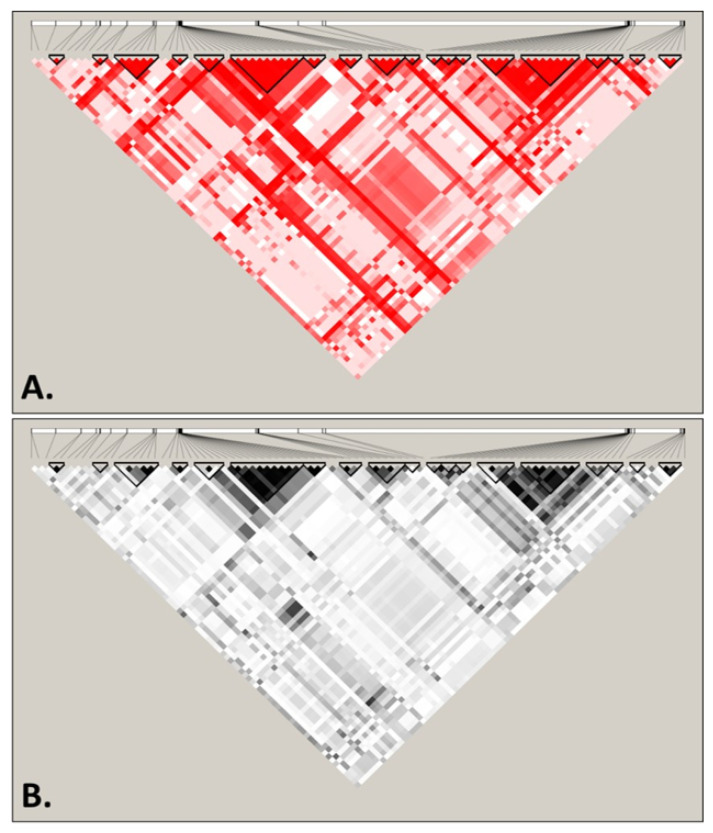
Patterns of linkage disequilibrium (LD) across the mtDNA genome of *Saccharomyces cerevisiae* displayed using *D’* (**A**) and *r*^2^ (**B**). Blocks of linkage disequilibrium are highlighted using black triangles. Analyses were performed considering 5475 bp across 6646 genomes.

The second strategy consisted of the use of Haploview software, a tool that calculates basic linkage disequilibrium (LD) statistics, highlighting blocks of linkage in the overall sequence. Linkage disequilibrium is useful for providing information on how a population is structured by identifying the associations between alleles, and to understand the existing recombination patterns in order to guide subsequent analyses. The results obtained for the 5475 bp across 6646 genomes, summarized in Figure 3, were obtained using *D’* (Figure 3A) and *r*^2^ (Figure 3B) statistics, both showing the LD pattern across the *S. cerevisiae* mitogenome for the analyzed SNP positions. Blocks with substantial LD are highlighted, suggesting that higher LD is only observed within genes (or exons), with the existence of haplotypic blocks (highlighted in the figure) in intragenic regions only. However, these blocks never extend across two genes, suggesting a permanent break of linkage occurring between genes, thus validating some of the subsequent analyses, since they assumed independence of SNPs. It is likely that the high concentration of AT base pairs throughout these regions, as well as the high number of repeats, is a major biochemical feature underlying the high recombination rate.

This high diversity, partially caused by recombination, results in a lack of general patterns when considering the phenotypic diversity of the yeast isolates, as discussed in the following sections. Emerging diversity due to mutational events is easily re-established in different backgrounds due to the high recombination rate. In technical terms, for the development of this work, the results showed that a great portion of the diversity is basically independent (low LD) in *S. cerevisiae* mtDNA, allowing the application of more robust statistical analyses.

### 3.3. Genetic Structure of S. cerevisiae Mitochondrial DNA

The general genetic structure of the mtDNA genome was investigated following three main methodologies, all based on clustering and genetic distances and therefore suitable for recombining systems: neighbor-joining trees, principal component analysis (PCA), and sparse non-negative matrix factorization (sNMF). Considering the steep recombination rate, and the lack of large haploblocks, as discussed in the previous section, it is clear that investigations based on the establishment of lineages and phylogenetic analyses, as currently used in phylogenetic and phylogeographic studies of higher eukaryotes, are inadequate [63]. We based our examination on the establishment of clusters that could agglomerate common diversity, highlighting geographic structuring or common source. The neighbor-joining tree, although it is a phylogenetic method, is based on genetic distances between sequences. PCA and sNMF, an individual ancestry estimator, also aim to establish patterns between sequences. The lack of LD throughout most of the molecule made these analyses feasible.

Considering our group of 6646 genomes, we searched databases in order to obtain geographical (country of origin) and technological/source information (what they are used for or where they were obtained from) for all *S. cerevisiae* strains for which mitogenomes were collected (Supplementary Data S1). We chose to include several isolates of the same strain in order to detect intra-strain mitogenome diversity, as found for the nuclear genome, following the same strategies as are used in populational analysis of other taxonomic groups. For 1948 yeast isolates, clear information regarding its technological applications was found. Regarding technological applications, data were divided into eight groups to facilitate categorization, in the same way as has been done before in similar works [64,65]: wine and vine (496 isolates), laboratory (452 isolates), natural environments—soil woodland, plants and insects (353 isolates), clinical (283 isolates), other fermented beverages (138 isolates), beer (70 isolates), sake (52 isolates), and bread (37 isolates). Regarding geographical origins of the isolates, 23 groups were created to categorize the 860 isolates for which geographical information was available in databases. A total of 646 yeast isolates were associated with data considering both geographical and technological information. In contrast, no information of any kind was found regarding 4751 isolates, mostly those available as raw data in the SRA database. Although these isolates were considered in the analyses performed, they were subsequently omitted from the visualizations in order to simplify group categorization.

#### 3.3.1. Principal Component Analysis (PCA)

**Figure 4:**
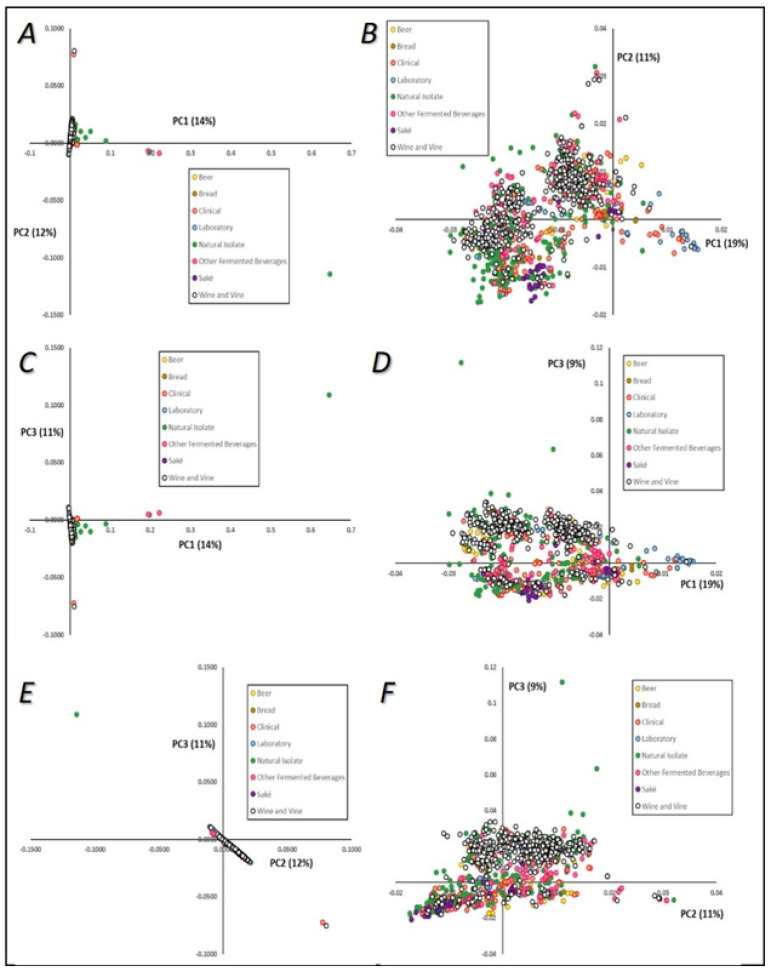
Principal component (PC) analysis visualization of the mtDNA genomes of *Saccharomyces cerevisiae*. Colors are indicative of strains’ technological applications. **A**—PC1 versus PC2; **B**—PC1 versus PC2, after exclusion of outliers defined in Panel **A**; **C**—PC1 versus PC3; **D**—PC1 versus PC3, after exclusion of outliers defined in Panel **C**; **E**—PC2 versus PC3; **F**—PC2 versus PC3, after exclusion of outliers defined in Panel **E**.

**Figure 5:**
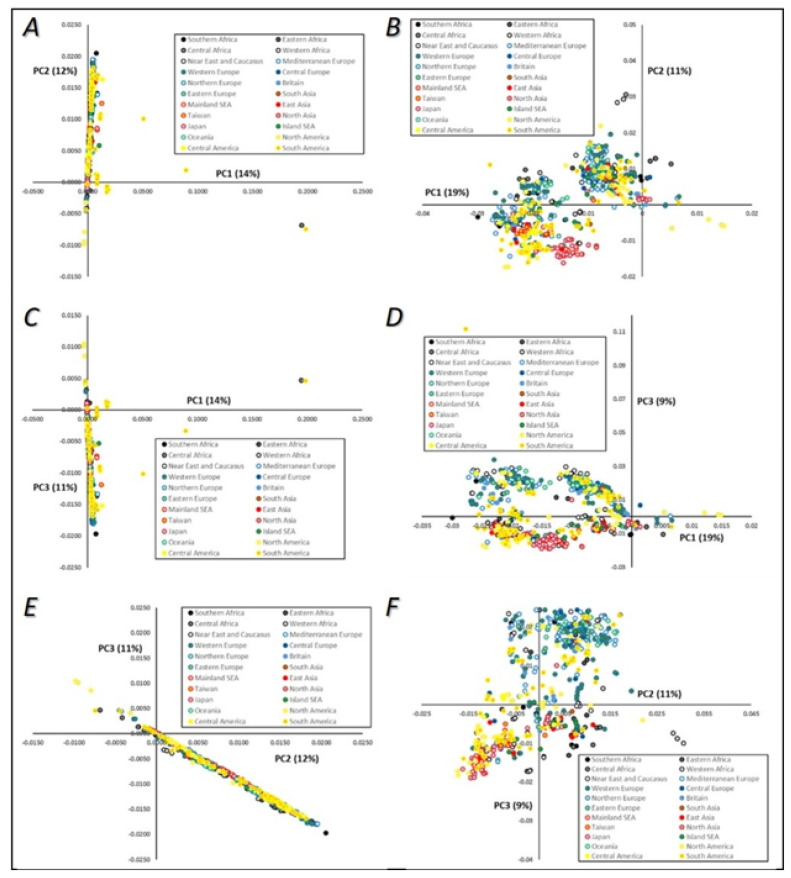
Principal component (PC) analysis visualization of the mtDNA genomes of *Saccharomyces cerevisiae*. Colors are indicative of strains’ geographical origins. **A**—PC1 versus PC2; **B**—PC1 versus PC2, after exclusion of outliers defined in Panel **A**; **C**—PC1 versus PC3; **D**—PC1 versus PC3, after exclusion of outliers defined in Panel **C**; **E**—PC2 versus PC3; **F**—PC2 versus PC3, after exclusion of outliers defined in Panel **E**.

The genetic diversity of our database was first assessed using principal component analysis, considering grouping both by technological source (Figure 4), and then by geographical origin of strains (Figure 5). The three major principal components (PCs), which explained in total 37% of the strains’ mtDNA genetic diversity, were plotted two by two. Although the cumulative explained variance was not very high, we used PCA visualization to understand patterns of segregation according to yeasts’ technological source or geographical origin. No clear patterns of genetic relatedness or clustering between strains sharing the same technological origin were evident (Figure 4A,C,E), although some samples were visible as outsiders to the major group of sequences, especially from the “other fermented beverages” and “natural source” categories, which might be expected to show higher diversity. When these outsiders were excluded, a higher resolution was obtained within the major group of isolates (Figure 4B,D,F). A clear division between two groups can be observed in Figure 4B, although both were composed by strains from common technological origins. These sub-groups were composed mainly of strains from the “wine and vine” subgroups, and separation was observed mainly by first and second principal components (Figure 4B), but also between first and third principal components (Figure 4D). An interesting result was that the upper-right group cluster mainly included strains from “beer” and “bread” sources (Figure 4B), while the bottom-left group clustered with strains from “saké” and with the majority of “natural” isolates. This could help to highlight the phenotypic diversity observed when analyzing *S. cerevisiae* strains, together with the profiles of their biotechnological products. In these two panels (Figure 4B,D) it can also be seen that some “laboratory” strains grouped together in the right part of the first principal component visualization, clustering with some clinical isolates and placed apart from strains from other origins, such as wine and vine strains, natural isolates, and strains obtained from fermented beverages.

When considering strains’ geographical origins (Figure 5), it seemed evident that there is very little geographic clustering as far as *S. cerevisiae* mtDNA diversity is concerned. While a few samples were placed as outliers, it is difficult to discern any major trend across PC1–PC3. Considering PCA location-wise, most data formed single clusters near the axis of the graphics (Figure 5A,C) with a small number of outliers corresponding to South America and Central African samples, although these exceptions could correspond to low-quality samples. In general, all samples were displayed within a continuous trend (gradients) established by the pairs of principal components. However, the general picture was that these samples did not display any type of geographic clustering and were placed along these gradients independently of geography. Single clusters were extended throughout one of the vectors, but this did not establish any type of geographic trend. Once again, similar geographical trends (or the absence of them) can be observed when analyzing yeast nuclear genomes, as will be discussed later.

#### 3.3.2. Sparse Non-Negative Matrix Factorization (sNMF)

**Figure 6:**
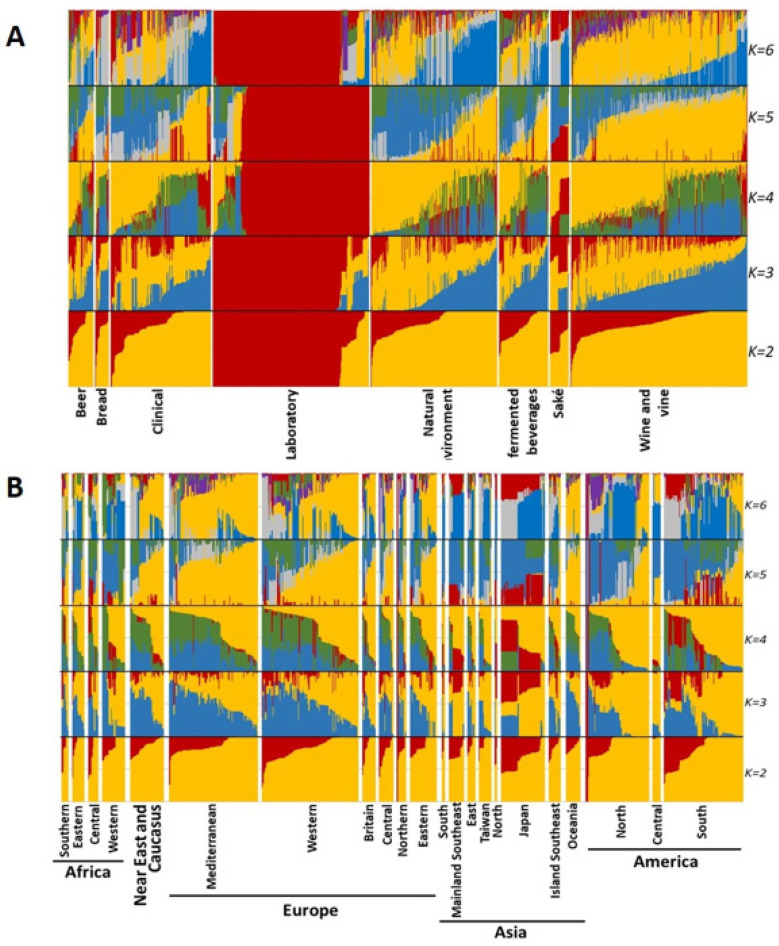
Individual ancestry estimates of *Saccharomyces cerevisiae* mtDNA genomes using the sNMF algorithm, considering strains categorized according to their technological application (**A**) or geographical origin (**B**). Individual mtDNA genetic diversity is clustered into genetic components that could represent ancestral *S. cerevisiae* mitogenome diversity. Analyses were performed considering the clustering of the data into two to six components that are represented in each analysis by different colors. The colors in each analysis were selected for a clear visual representation of each component.

Figure 6 displays the results of sNMF analysis, considering between two and six components and separating samples by technological source (Figure 6A) and by geographical origin (Figure 6B). sNMF results were complementary to the ones obtained by PCA. When examining grouping by technological source (Figure 6A), at K = 2, laboratory strains displayed a clearly different pattern from other samples, which was also maintained for higher K values. In PCA, the laboratory samples also mostly clustered together. Another interesting result is that for K = 3 to K = 6, the samples from “saké” seemed to display two different major genetic profiles, each with different proportions of the components. At K = 6, the “wine and vine” group displayed a high frequency of a major component (yellow) that was less frequent in some other groups, suggesting some deeper level of clustering.

Regarding strains’ geographical origins (Figure 6B), a very similar situation was observed in sNMF results. For K = 2, no clear pattern was visible. When considering K = 3, there seemed to exist some preponderance of the blue component in the analysis of European and African groups, while Asia showed a higher frequency of the red component. K = 4 patterns were less discernible and at K = 5, again, the yellow component seemed to be more frequent in Europe (and the associated Russia and Near East) and it was the most prominent feature in the analysis (maintained for K = 6). The blue component was mostly displayed in Asia. Nevertheless, the patterns were very mild and the geographical clustering of *S. cerevisiae* mtDNA seems virtually nonexistent.

#### 3.3.3. Neighbor-Joining Phylogenetic Trees

Phylogenetic analysis showed no clear separation between the groups considered, with small exceptions, as expected when considering results within such a complex structure (Supplementary Data S4). Neighbour-joining phylogenetic trees based on pairwise SNP differences in the alignments were generated in order to find clusters considering strains’ technological applications (Supplementary Figure S4A) and geographical origin (Supplementary Figure S4B). When considering strains’ origins in terms of technological application, although no clear separation was observed, some tenuous patterns were identified: (i) some laboratory strains (marked in blue) clustered together in a single branch in the left part of the dendogram; (ii) some well-defined sub-clades of strains from wine and vine origins (white circles) clustered together; (iii) clinical isolates (orange) did not group with each other, but instead were spread throughout all tree. This trend was expected, since clinical strains are mostly isolates from other sources that gained virulence, and this is also observed when analyzing full nuclear genomes.

When considering the geographic provenances of the isolates, the neighbor-joining tree was extremely interleaved and intertwined between different geographies with no patterns of relevance, as also revealed also by the other analysis described above.

### 3.4. Population Structure Based on Strains’ Technological Sources and Geographical Origins

Population structure was assessed in order to further evaluate the existence of patterns of divergence among subpopulations catalogued using strains’ technological applications or geographical origins. *Fst* values were calculated as explained in the Materials and Methods section, and a NJ tree was constructed for each type of cataloguing method.

**Figure 7:**
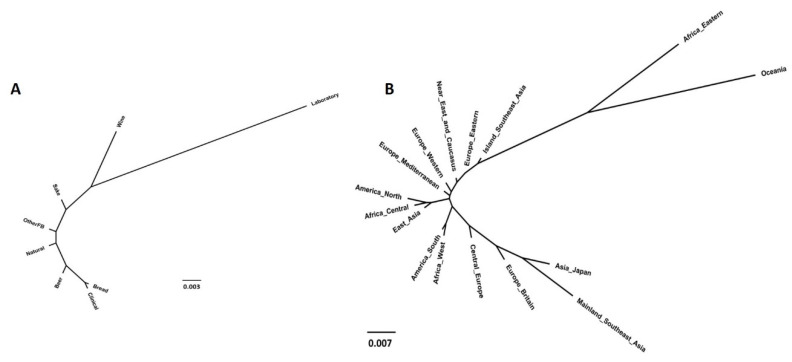
Population structure of *Saccharomyces cerevisiae* obtained using the neighbor-joining tree estimated from the matrix of *Fst* values computed by comparing groups of strains according to their technological applications (**A**) and geographical origins (**B**).

Regarding strains’ technological origins, among all tested combinations, higher *Fst* values (Table 2) were obtained when comparing laboratory strains with strains from all the other sources (0.0281 < *Fst* < 0.0666), which already correspond to a scenario of moderate genetic differentiation (0.05 to 0.15) according to Hartl and Clark [66]. The lowest *Fst* values were obtained when comparing strains used to produce beer with strains from natural origins and from other fermented beverages (*Fst* values of 0.0003 and 0.0009, respectively). These results were validated by the general statistics obtained for this analysis and shown in Supplementary Data S2. The NJ tree (Figure 7) was in accordance with these data, showing a high distance of laboratory strains from the remaining groups, as was expected (discussed later).

When analyzing population structures and categorizing strains per geographical origin (Table 3, Figure 7B, Supplementary Data S3), similarly to other methods already shown, no clear conclusion could be drawn. However, some peculiar results can be highlighted: (i) strains from Oceania showed the highest *Fst* values (0.01 < *Fst* < 0.19) when compared with strains from other provenances, some of them corresponding to high genetic differentiation (*Fst* > 0.15), and the majority to a scenario of moderate genetic differentiation (*Fst* between 0.05 and 0.15) [66]; (ii) “Africa Eastern” also showed some separation from other groups (average *Fst* of 0.023); (iii) on the other extreme in relation to “Oceania” and “Africa Eastern” were the grouped strains from “Mainland Southeast Asia”, which showed a moderate genetic differentiation from almost all other groups. Given the lack of major patterns observed in the previous analyses, these results were expected.

## 4. Discussion

*Saccharomyces cerevisiae* is the microorganism per excellence in biotechnological research, mainly due to its diverse phenotypic heterogeneity, and it is used in an increasing number of industrial applications, such as wine, bread, beer, saké, etc. Although, for a long period, this yeast has been mostly associated only with human-related activities, it is now known that isolates from natural sources represent a very important group, both ecologically and in terms of further scientific potential, which has led to an increase of the number of studies comparing groups of strains from different technological and/or geographical origins [67]. The paths of evolution that this yeast has followed over many decades are still somehow unexplored, and their complete understanding will allow the impact of indigenous populations on several industrial applications to be evaluated, improving the final products and leading to the discovery of new ones.

Currently, the number of studies focusing on yeast mtDNA is small, especially when comparing its diversity with strains’ phenotypic or biotechnological data. Even studies directed at yeast genomics tend to completely exclude this molecule from their analyses. A likely reason for this could be the extreme difficulty encountered in obtaining comparable mitogenomes, even when using the most sophisticated alignment tools. The great number of adenine and thymine base pairs, the extension of the intergenic sequences, and the fluctuating size of the genome and its variable gene content cause difficulties in these analyses. In this regard, as far as we know, our work is pioneering in its focus on this molecule, combining a very large dataset collected from publicly available databases, including available strains’ information. This work is also the first one, again to the extent of our knowledge, to establish a pipeline by which to analyze yeast mtDNA, bypassing the exposed difficulties, which opens the door to new studies focusing on yeast mitogenomes. Around 80 species within the Saccharomycotina subphylum have mitogenome sequences available, revealing a large diversity in their structure and organization [68]. In particular, the work of Sulo et al. [48] allowed the mitogenome variation across several *Saccharomyces* species to be understood, revealing important features such as species-specific alterations in gene order. However, a higher number of strains should be included, particularly including less known species, in order to deeply understand the particular relation between mito- and nuclear genomes. With the approaches suggested in the present work, we expect that many more *Saccharomyces* species and strains will have their mitogenomes analyzed in the future.

Data used in the present study were obtained from online databases, particularly from the publicly available genetic database of the NCBI (National Center for Biotechnology Information Search database). The data were obtained in two formats: fasta sequences of the available complete mtDNA of *S. cerevisiae* deposited in the “nucleotide” section of NCBI (these data are completely annotated in terms of genes), and raw next-generation sequencing files from genomic data (DNA) of *S. cerevisiae* deposited in the Sequence Reads Archive of NCBI (SRA). Using 184 *S. cerevisiae* mitochondrial DNA sequences that were fully annotated in terms of genes, proteins, and associated functions, we defined a reference comparable sequence between all mtDNA genomes. This database proved to be a useful resource for in different mitogenomic analyses, allowing the mentioned problems related with genome size and base and gene content to be overcome. As we gathered the mitochondrial DNA from the SRA section of the NCBI database, we also obtained the nuclear DNA of those same individuals. As a result, we will be able to perform further studies similar to this one, but focusing on the remaining genome, which will allow us to corroborate/contrast the results of this study.

Early published work on *S. cerevisiae* mtDNA failed to discover the recombination in this molecule, which was not considered before the discovery that *S. cerevisiae* mtDNA is biparentally inherited, in opposition to higher eukaryotes [24]. It has been widely accepted for a long time that recombination of the nuclear genome of *S. cerevisiae* occurs in nature at a high rate [23], but it is now recognized that the recombination rate in mtDNA is higher than that in the nuclear genome [17]. In our study, high recombination rates were detected in the analyzed yeasts’ mitogenomes, considering the extracted sequence of 5475 bp across 6646 genomes, using PHASE software and LD measures. Currently, it is general knowledge that recombination plays a very important role in the evolution of the genome, not only instigating a very high haplotypic diversity through new combinations of existing variants, but also by promoting a faster disruption of newly formed haplotypes, as also detected in this study. High numbers of recombination events throughout the mitochondrial molecule could also be responsible for the instability in the gene content across mitogenomes of different strains, as occurs in processes similar to known cases for large deletions and gene conversions observed in the human genome [59].

Several methods were used to understand the genetic structure of the *S. cerevisiae* mitogenome, in particular principal component analysis, sparse non-negative matrix factorization, and neighbor-joining phylogenetic trees associated with statistical analyses. Considering the geographical origins of the isolates, no relevant patterns were discovered, as expected. This result was concordant with our previous work [64] considering the nuclear genomes of *S. cerevisiae,* in which no relevant relations were obtained between strains’ geographical origins and their genetic data. A different scenario was obtained in both works when considering the technological origins of the isolates. One highlighted result obtained in the present work was related to laboratory strains, which already showed moderate differentiation from strains from other groups (0.0281 < *Fst* < 0.0666). These results were somewhat expected, since yeasts used in laboratory applications undergo several mutations according to years and years of laboratory use, which leads to some adaptation and evolution. This was shown previously by several authors, but they focused only on nuclear genomic data [69,70,71,72,73,74,75]. Another important result was the separation, to some extent, of wine strains, which could justify an already adaptive evolution to a very specific biotechnological niche. Again, using nuclear genomic data, this adaptation was previously shown for wine yeasts, even within the same strain, with the detection of microevolutionary changes when adapting to vineyard ecosystems [76]. The observed heterogeneity and the profiles obtained when cataloguing strains by isolation source were also in accordance with the key results obtained by other researchers inferred from full nuclear genomes [1,36]. In these publications, authors also detected domestication of some strain groups, which was concluded to be associated with improved fermentation properties of those isolates. It is our understanding that this is the first time that such type of conclusions have been drawn from mitogenomes, which greatly simplify the process of sequencing and data analysis due to the reduction of genome size and the high mutation and recombination rates being reflected in a great discriminative power of strain identification for a small analyzed sequence. The high number of mtDNA copies compared with the nuclear genome also allows typing of the strains even with lower amounts of genetic material, including in forensic and archaeological settings. This feature, together with the high individual discrimination that mtDNA allows, could prove very important for the former field.

Although used to infer phylogenetic relationships among species, mtDNA phylogenies often reveal variations from those generated using nuclear genomes [77], prompting further important research regarding this molecule and highlighting the need to expand mitogenomic research to a broad range of yeast species, in order to understand the evolutionary forces behind these differences. In addition, intraspecific analyses, such as the ones presented in this work, are needed to provide the assessment of mitogenome variation in terms of organization, topology, and diversity within different strains of the same species, and also within different isolates of a same strain. This information, when added to evolutionary trees obtained using nuclear genomes, will allow the full evolutionary patterns of yeast to be obtained. In addition, the method developed here, consisting of the design of a reference sequence comparable between all mitogenomes, will allow a large number of yeast mitogenomes to be analyzed and compared. This will allow the final conclusion, related to a large group of strains, that the mitogenome interplays with the nuclear genome to reveal increased phenotypic variation, in contrast to the lack of population structure (and lack of connection with nuclear genomic studies) reported in previous studies analyzing yeast mitogenomes.

## Figures and Tables

**Table 1:** General statistics obtained for the final dataset of 6646 *Saccharomyces cerevisiae* mtDNA genomes using Arlequin and DNAsp.

General Parameter	Statistics
Number of sequences	6646
Size (bp)	5475
Number of polymorphic sites	526
Number of haplotypes	1265
Number of observed transitions	273
Number of observed transversion	291
Nucleotide composition (%)—C	13.09
Nucleotide composition (%)—T	41.33
Nucleotide composition (%)—A	30.95
Nucleotide composition (%)—G	14.63
Gene diversity	0.9665 ± 0.0032
Mean number of pairwise differences	38.632869 ± 16.797110
Nucleotide diversity (average over loci)	0.007354 ± 0.003535
Tajima’s *D p*-value	0.10600
Fu’s *FS p*-value	0.59400

**Table 2:** *Fst* statistics calculated using DNAsp between each group of strains, categorized according to their technological source, using mtDNA genomic data. Values higher than 0.01 are highlighted. Other Fb—other fermented beverages.

Beer	Bread	Clinical	Laboratory	Natural	Other Fb	Saké	Wine and Vine	
-	0.0031	0.0034	0.0543	0.0031	0.0009	0.0037	0.0025	**Beer**
	-	0.0016	0.0545	0.0075	0.0127	0.0097	0.0025	**Bread**
		-	0.0666	0.0035	0.0073	0.0133	0.0056	**Clinical**
			-	0.0374	0.0312	0.0281	0.0235	**Laboratory**
				-	0.0003	0.0024	0.0028	**Natural**
					-	0.0028	0.0038	**Other Fb**
						-	0.0020	**Saké**
							-	**Wine and vine**

**Table 3:** *Fst* statistics calculated using DNAsp between each group of strains, categorized according to their geographical origins, using mtDNA genomic data. Values higher than 0.02 are highlighted.

Africa_Central	Africa_Eastern	Africa_West	America_North	America_South	East_Asia	Island_Southeast_Asia	Asia_Japan	Mainland_Southeast_Asia	Europe_Britain	Central_Europe	Europe_Eastern	Europe_Mediterranean	Europe_Western	Near_East_and_Caucasus	Oceania	
-	0.0238	0.0127	0.0079	0.0054	0.0054	0.0222	0.0033	0.0268	0.0459	0.0215	0.0083	0.0152	0.0181	0.0162	0.0769	**Africa_Central**
	-	0.0263	0.0078	0.0002	0.0342	0.0316	0.0184	0.0484	0.0229	0.0179	0.0299	0.0185	0.0184	0.0295	0.0154	**Africa_Eastern**
		-	0.0162	0.0012	0.0164	0.0120	0.0046	0.0322	0.0209	0.0245	0.0100	0.0150	0.0115	0.0170	0.0888	**Africa_West**
			-	0.0107	0.0091	0.0202	0.0452	0.0733	0.0018	0.0137	0.0252	0.0198	0.0186	0.0116	0.0904	**America_North**
				-	0.0149	0.0203	0.0067	0.0303	0.0129	0.0118	0.0220	0.0026	0.0069	0.0126	0.1132	**America_South**
					-	0.0195	0.0383	0.0779	0.0031	0.0197	0.0211	0.0028	0.0073	0.0163	0.0460	**East_Asia**
						-	0.0006	0.0247	0.0352	0.0224	0.0104	0.0177	0.0172	0.0109	0.0459	**Island_Souhteast_Asia**
							-	0.0082	0.0026	0.0185	0.0239	0.0098	0.0121	0.0344	0.1680	**Asia_Japan**
								-	0.0123	0.0071	0.0391	0.0339	0.0390	0.0638	0.1946	**Mainland_Southeast_Asia**
									-	0.0164	0.0003	0.0218	0.0189	0.0175	0.0921	**Europe_Britain**
										-	0.0167	0.0156	0.0146	0.0033	0.1049	**Central_Europe**
											-	0.0031	0.0031	0.0060	0.0424	**Europe_Eastern**
												-	0.0056	0.0031	0.1090	**Europe_Mediterranean**
													-	0.0062	0.0892	**Europe_Western**
														-	0.0490	**Near_East_and_Caucasus**
															-	**Oceania**

## Supplementary Materials

The following are available online at https://www.mdpi.com/2076-2607/8/7/1001/s1, Supplementary File S1: accession number, strain code, technological group and geographical origin of all the samples used in the present study. Supplementary File S2: General statistics obtained for 1864 *Saccharomyces cerevisiae* mtDNA genomes, using Arlequin and DNAsp, considering strains categorization according to their technological application. Supplementary File S3: General statistics obtained for 1864 *Saccharomyces cerevisiae* mtDNA genomes, using Arlequin and DNAsp, considering strains categorization according to their geographical origin. Supplementary data S4: Neighbour-joining trees of Saccharomyces cerevisiae mitochondrial genomes. Colors are indicative of strains´ technological application (A) or geographical origin (B)
