# Supplementary material for: Population Analysis and Evolution of Saccharomyces cerevisiae Mitogenomes
Source: Microorganisms. 2020 Jul 4;8(7):1001. doi: 10.3390/microorganisms8071001 (PMC7409325; doi:10.3390/microorganisms8071001)
Supplement: Supplementary file 1 [file microorganisms-08-01001-s001.zip › Supplementary Data/Supplementary Data S2.docx]

**Supplementary Data S2**

General statistics obtained for 1864 *Saccharomyces cerevisiae* mtDNA genomes, using Arlequin and DNAsp, considering strains categorization according to their technological application.

Other FB – other fermented beverages.

Underlined values correspond to statistically significance p-values (p≤0.05).

| **Group** | **N** | **Number of haplotypes** | **Haplotype diversity, Hd** | **Average number of differences, K** | **Nucleotide diversity, Pi** | **Tajima's D** | **Statistical significance (p-value)** | **Fu and Li's D** | **Statistical significance (p-value)** | **Fu and Li's F** | **Statistical significance (p-value)** |
| --- | --- | --- | --- | --- | --- | --- | --- | --- | --- | --- | --- |
| **Beer** | 70 | 15 | 0,68696 | 1,04928 | 0,00032 | 0,75979 | >0.1 | 0,89327 | >0.1 | 1,00731 | >0.1 |
| **Bread** | 37 | 6 | 0,41742 | 0,45646 | 0,00014 | 0,85242 | >0.1 | 0,34800 | >0.1 | 0,62208 | >0.1 |
| **Clinical** | 281 | 19 | 0,48742 | 0,66075 | 0,00020 | 0,42238 | >0.1 | -0,33425 | >0.1 | 0,02714 | >0.1 |
| **Laboratory** | 438 | 3 | 0,01818 | 0,01822 | 0,00001 | -1,24432 | >0.1 | **2,23893** | **<0.02** | 0,81217 | >0.1 |
| **Natural** | 354 | 34 | 0,62766 | 1,18790 | 0,00036 | -1,33558 | >0.1 | -1,09173 | >0.1 | -1,45087 | >0.1 |
| **Other FB** | 138 | 21 | 0,61187 | 1,37396 | 0,00042 | -0,29648 | >0.1 | -1,53633 | >0.1 | -1,17455 | >0.1 |
| **Sake** | 52 | 13 | 0,49548 | 0,89517 | 0,00027 | -0,04467 | >0.1 | -1,53633 | >0.1 | -1,17455 | >0.1 |
| **Wine** | 494 | 31 | 0,48510 | 0,80445 | 0,00024 | **-1,81012** | **<0.05** | -1,53633 | >0.1 | -1,17455 | >0.1 |
