# Supplementary material for: Population Analysis and Evolution of Saccharomyces cerevisiae Mitogenomes
Source: Microorganisms. 2020 Jul 4;8(7):1001. doi: 10.3390/microorganisms8071001 (PMC7409325; doi:10.3390/microorganisms8071001)
Supplement: Supplementary file 1 [file microorganisms-08-01001-s001.zip › Supplementary Data/Supplementary Data S3.docx]

**Supplementary Data S3**

General statistics obtained for 1864 *Sacharomyces cerevisiae* mtDNA genomes, using Arlequin and DNAsp, considering strains categorization according to their geographical origin.

Statistical parameters were only calculated to groups with more than 10 isolates.

Underlined values correspond to statistically significant *p*-values (*p* ≤ 0.05).

| **Group** | **N** | **Number of haplotypes** | **Haplotype diversity, Hd** | **Average number of differences, K** | **Nucleotide diversity, Pi** | **Tajima's D** | **Statistical significance (p-value)** | **Fu and Li's D** | **Statistical significance (p-value)** | **Fu and Li's F** | **Statistical significance (p-value)** |
| --- | --- | --- | --- | --- | --- | --- | --- | --- | --- | --- | --- |
| **Africa_Central** | 13 | 8 | 0,85897 | 2,66667 | 0,00064 | 0,33643 | >0.1 | 0,45235 | >0.1 | 0,48233 | >0.1 |
| **Africa_Eastern** | 14 | 9 | 0,83516 | 1,81319 | 0,00044 | 0,58960 | >0.1 | 0,64909 | >0.1 | 0,72726 | >0.1 |
| **Africa_South** | 8 | 6 | 0,92857 | 3,07143 | 0,00074 | - | - | - | - | - | - |
| **Africa_West** | 26 | 13 | 0,84000 | 2,00615 | 0,00048 | 0,88071 | >0.1 | 1,06384 | >0.1 | 1,18366 | >0.1 |
| **America_Central** | 9 | 6 | 0,88889 | 2,00000 | 0,00048 | - | - | - | - | - | - |
| **America_North** | 72 | 19 | 0,66901 | 1,20853 | 0,00029 | 0,97726 | >0.1 | 0,47444 | >0.1 | 0,81301 | >0.1 |
| **America_South** | 90 | 28 | 0,75306 | 2,42397 | 0,00058 | **-1,93589** | **<0.05** | -1,39993 | >0.1 | -1,93797 | >0.05 |
| **East_Asia** | 23 | 12 | 0,86957 | 1,68775 | 0,00041 | 0,80923 | >0.1 | 0,51623 | >0.1 | 0,71337 | >0.1 |
| **Island_Southeast_Asia** | 14 | 9 | 0,87912 | 2,29670 | 0,00055 | 0,38349 | >0.1 | 0,01221 | >0.1 | 0,13151 | >0.1 |
| **Asia_Japan** | 50 | 22 | 0,85143 | 2,28980 | 0,00055 | 0,13234 | >0.1 | 0,35448 | >0.1 | 0,32472 | >0.1 |
| **Mainland_Southeast_Asia** | 17 | 8 | 0,72794 | 1,88235 | 0,00045 | 0,86496 | >0.1 | 0,73479 | >0.1 | 0,89580 | >0.1 |
| **Europe_Britain** | 15 | 7 | 0,72381 | 1,60000 | 0,00039 | 0,66909 | >0.1 | 0,51031 | >0.1 | 0,63983 | >0.1 |
| **South_Asia** | 4 | 3 | 0,83333 | 1,00000 | 0,00024 | - | - | - | - | - | - |
| **North_Asia** | 3 | 2 | 0,66667 | 1,33333 | 0,00032 | - | - | - | - | - | - |
| **Central_Europe** | 16 | 11 | 0,93333 | 2,66667 | 0,00064 | 0,55756 | >0.1 | 0,58596 | >0.1 | 0,66831 | >0.1 |
| **Europe_Eastern** | 30 | 17 | 0,91264 | 2,12414 | 0,00051 | 1,16234 | >0.1 | 0,66757 | >0.1 | 0,98556 | >0.1 |
| **Europe_Mediterranean** | 101 | 26 | 0,76931 | 1,52990 | 0,00037 | 0,51293 | >0.1 | -0,01713 | >0.1 | 0,24936 | >0.1 |
| **Europe_North** | 10 | 8 | 0,93333 | 1,82222 | 0,00044 | - | - | - | - | - | - |
| **Europe_Western** | 110 | 32 | 0,83169 | 1,95813 | 0,00047 | 1,24626 | >0.1 | **1,55021** | **<0.05** | 1,70308 | >0.05 |
| **Near_East_and_Caucasus** | 39 | 17 | 0,85830 | 1,83806 | 0,00044 | 0,84339 | >0.1 | 0,65053 | >0.1 | 0,85629 | >0.1 |
| **Oceania** | 16 | 8 | 0,84167 | 1,65833 | 0,00040 | -0,20198 | >0.1 | 0,14454 | >0.1 | 0,14454 | >0.1 |
