# Supplementary material for: Population Analysis and Evolution of Saccharomyces cerevisiae Mitogenomes
Source: Microorganisms. 2020 Jul 4;8(7):1001. doi: 10.3390/microorganisms8071001 (PMC7409325; doi:10.3390/microorganisms8071001)

**Supplementary data S4**

Neighbour-joining trees of *Saccharomyces cerevisiae* mitochondrial genomes. Colors are indicative of strains´ technological application (A) or geographical origin (B).


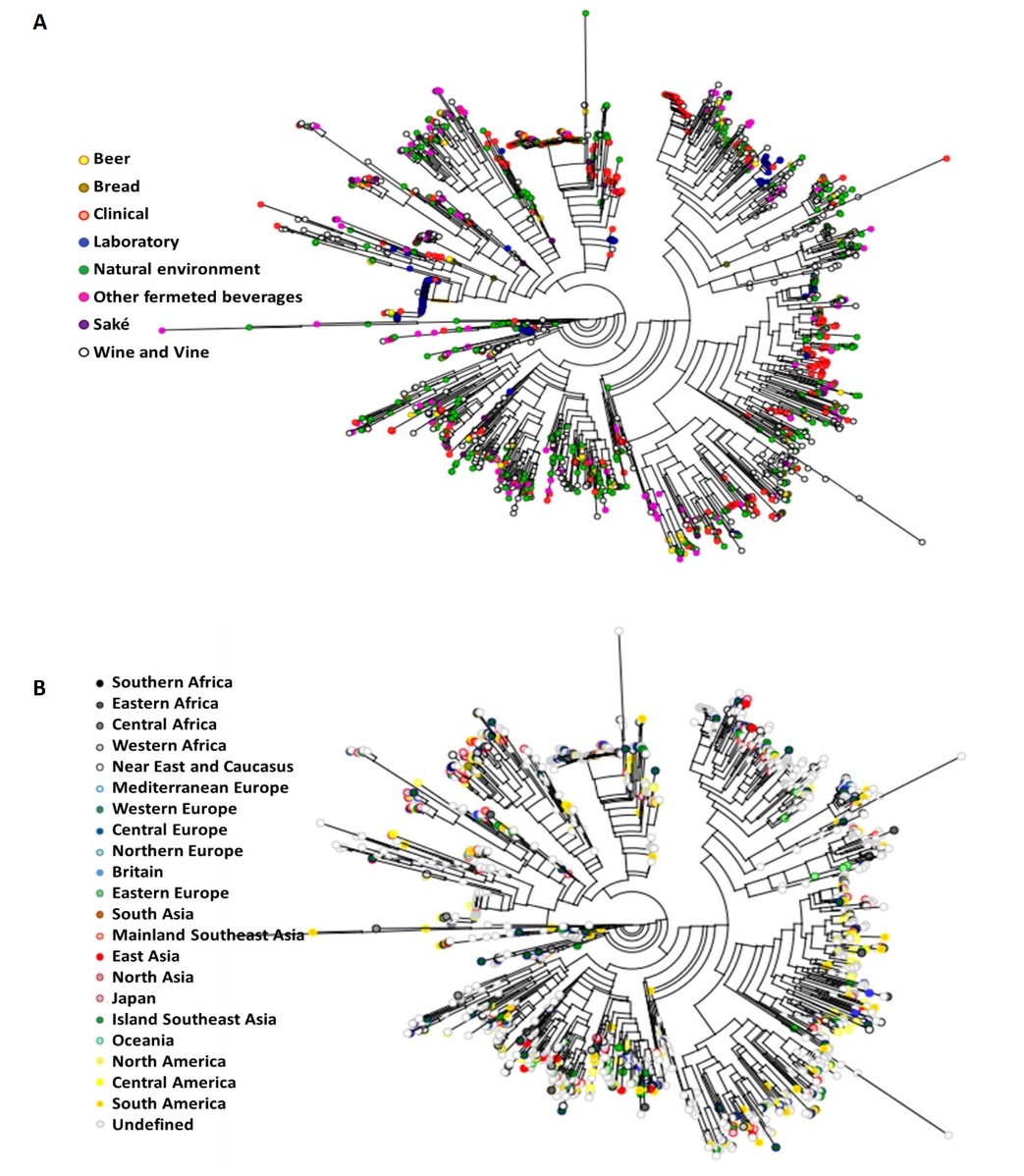

Supplement: Supplementary file 1 [file microorganisms-08-01001-s001.zip › Supplementary Data/Supplementary Data S4.docx]
